# Pt-TiO_2_/MWCNTs Hybrid Composites for Monitoring Low Hydrogen Concentrations in Air

**DOI:** 10.3390/s120912361

**Published:** 2012-09-10

**Authors:** Stefano Trocino, Andrea Donato, Mariangela Latino, Nicola Donato, Salvatore Gianluca Leonardi, Giovanni Neri

**Affiliations:** 1 Department of Mechanics and Materials, University of Reggio Calabria, 89124 Reggio Calabria, Italy; E-Mails: stefano.trocino@unirc.it (S.T.); andrea.donato@unirc.it (A.D.); 2 Department of Chemical Science and Technologies, University of Rome Tor Vergata, 00133 Roma, Italy; E-Mail: mariangela.latino@uniroma2.it; 3 Department of Matter Physics and Electronic Engineering, University of Messina, 98166 Messina, Italy; E-Mail: ndonato@unime.it; 4 Department of Industrial Chemistry and Materials Engineering, University of Messina, 98166 Messina, Italy; E-Mail: leonardis@unime.it

**Keywords:** hydrogen sensor, TiO_2_, MWCNTs, hybrid nanocomposites

## Abstract

Hydrogen is a valuable fuel for the next energy scenario. Unfortunately, hydrogen is highly flammable at concentrations higher than 4% in air. This aspect makes the monitoring of H_2_ leaks an essential issue for safety reasons, especially in the transportation field. In this paper, nanocomposites based on Pt-doped TiO_2_/multiwalled carbon nanotubes (MWCNTs) have been introduced as sensitive materials for H_2_ at low temperatures. Pt-TiO_2_/MWNTs nanocomposites with different composition have been prepared by a simple wet chemical procedure and their morphological, microstructural and electrical properties were investigated. Resistive thick-film devices have been fabricated printing the hybrid nanocomposites on alumina substrates provided with Pt interdigitated electrodes. Electrical tests in air have shown that embedding MWCNTs in the TiO_2_ matrix modify markedly the electrical conductivity, providing a means to decrease the resistance of the sensing layer. Pt acts as a catalytic additive. Pt-TiO_2_/MWNTs-based sensors were found to be sensitive to hydrogen at concentrations between 0.5 and 3% in air, satisfying the requisites for practical applications in hydrogen leak detection devices.

## 1. Introduction

In the past decades gas sensor devices have attracted widespread attention due to their potential application in environmental pollution monitoring, flammable and toxic gas detection and food quality control [1]. A chemical sensor can be defined as “a small device that as a result of a chemical interaction or process between the analyte and the sensor device transforms chemical or biochemical information of a quantitative or qualitative type into an analytically useful (electronic) signal”. Resistive chemical sensors, devices in which the electronic signal is represented by the electrical resistance (Ω) variation under different concentrations of the target gas, suffer from long-term drift and generally present poor selectivity, which could prevent their use when the detection of target is required in a mixture of various gaseous components [2]. Despite these drawbacks, they are largely employed today due to their simple use and low cost.

Hydrogen is a colourless, odourless and flammable gas at concentrations higher than 4%, Nowadays hydrogen is widely used in the chemical, petrochemical and semiconductor industry, and it is expected to gain great importance as a clean fuel in automotive and domestic fuel cells in the near future. The fabrication of reliable, robust and low cost sensitive sensors for the detection and monitoring of hydrogen is therefore a key issue in the development of hydrogen-based technologies. One critical aspect for the safe and efficient deployment of hydrogen is the ability of chemical sensors to meet the required performance specification for the growing hydrogen infrastructure [3].

In this regard, the aims of this work are to synthesize TiO_2_/MWCNTs hybrid nanostructures and test their hydrogen sensing capability at low temperature. The research on carbon nanotubes (CNTs) and their applications has been attracting a great deal of attention because of their individual and interesting properties for practical applications. Carbon nanotubes exhibit remarkable structural and physical properties that make them potentially useful for applications including nano-electronics, multifunctional composite materials, field emission devices, *etc*. Their high surface area, size and hollow geometry also make them promising candidates for use as active sensing elements in chemical sensors. Among the variety of the nanomaterials, hybrid nanostructured metal oxide/carbon nanotube composites are believed to provide superior performances, exhibiting synergistic effects between the metal oxide and carbon nanotube phase [4–6]. On the other hand, TiO_2_ is a metal oxide which can be prepared with high specific surface, abundant surface-active groups, and presents good chemical stability. These characteristics make titania a valuable material for gas sensing. June *et al.* reported the high sensitivity of TiO_2_ thin films to hydrogen, in reducing or inert atmospheres, operating at 300 °C [7]. Paulose *et al.* showed that highly-ordered titania nanotube have excellent response to hydrogen gas at room temperature, reporting a change in electrical resistance to 1,000 ppm hydrogen of more than eight orders of magnitude [8].

Additionally, Ti atoms on the surface of nanotitania have coordinative affinity for the oxygen of the carboxylic group of functionalized CNTs. Thus, nanocomposites consisting of CNTs coated with TiO_2_ may induce interesting charge transfer and enhance the sensitivity due to the excellent electronic property of nano TiO_2_ and CNTs [9].

The ability of a CNT/metal oxide composite sensor to detect the presence of chemicals relies on the interaction between gas molecules and the surface of the sensing films. This interaction is affected by many factors such as the temperature of operation, the gas being analyzed, the sensor geometry and packaging. Gas detection is enabled by a change in the electric resistance arising from a surface phenomenon. The reactivity of a surfaces is dependent on its characteristics that is, on its elemental composition including any doping, or impurity constituents, on its electronic and defect structure and on its microstructure. Changes in the surface characteristics of the active layer can induce a change on the sensor performance. It has been reported that improvement of the sensing properties (selectivity and sensitivity) of metal oxides can be achieved by the addition of small amounts of noble metals to the active layer. Metal additives, such as Pd and Pt, are dispersed on the oxide as activators or sensitizers to improve the gas selectivity and to lower the operating temperature [10]. Two mechanisms have been suggested to explain the effects of noble metals on sensing properties: electronic and chemical sensitization [11]. The electronic sensitization, typical for Pd, considers that there are partially oxidized metallic clusters which are electronically connected to the supporting metal-oxide (pinning of the Fermi-level). The reaction with the target gases changes the stoichiometry/chemical state of the noble metal clusters and therefore the changes of the position of the Fermi level are translated into sensor resistance changes. In the chemical sensitization, typical for Pt, the action of noble metal, which is expected to form metallic clusters at the surface of the metal oxide, is to improve the gas-semiconductor reaction by a catalytic effect. These clusters on the semiconductor surface have a higher sticking coefficient to gasses than metal oxide, and dissociate nearly all the gas molecules, spilling the products over the semiconductor surface. TiO_2_/MWCNTs doped with Pt have been then also investigated as 3-component hybrid composites in order to benefit from the possible Pt catalytic promoter effect on hydrogen sensing in air.

## 2. Experimental Section

### 2.1. Sample Preparation

Before use, MWCNTs (Aldrich) were functionalized by a heat treatment in concentrated nitric acid (15 M) in refluxing for 18 hours at 110 °C to modify their surface. MWCNTs were rinsed with distilled H_2_O until the pH of the solution was neutral, and finally dried at 80 °C in a vacuum oven.

TiO_2_/MWCNTs composites with different nominal C/Ti molar ratio were prepared by a modified sol-gel method previously reported by Jitianu [12], dispersing MWCNTs in a solution of titanium isopropoxide in isopropanol previously held under reflux at 80 °C for 1 h. Then, the suspension was stirred with a magnet for 3 h, and finally the TiO_2_/MWCNTs composites were separated from the solution by filtration. The samples were treated at 400 °C for 2 h in air. Pt-doped composites with a nominal Pt loading of 2%wt were prepared by wet impregnation of TiO_2_/MWCNTs samples, with a solution of platinum acetylacetonate in acetone. Pt-TiO_2_/MWCNTs samples were then treated at 200 °C for 2 h in a mixture of 5% H_2_ in argon. The main characteristics of the different Pt-doped and as prepared TiO_2_/MWCNTs composites are reported in Table 1.

### 2.2. Sample Characterization

XRD measurements on powdered samples were performed using a Philips X-Pert diffractometer equipped with a Ni β-filtered Cu-Kα radiation at 40 kV and 20 mA. Data were collected over a 2θ range of 10–85°, with a step size of 0.05° at a speed of 0.05° s^−1^. Diffraction peak identification was performed on the basis of the JCPDS database of reference compounds [13]. The mean particle size was calculated from line broadening analysis of the diffraction peaks by using the Scherrer Equation (1):

(1) $$\langle D\rangle =\frac{0.9\lambda }{B\cos\theta }$$

where λ, B and θ are the x-ray wavelength of the radiation used (CuK_α1_ = 1.54056 Ǻ), the full width at half maximum (FWHM) of the diffraction peak (radians) corrected for the instrumental broadening, and the Bragg diffraction angle (degrees), respectively.

The morphology of the samples was investigated by SEM, using a Philips XL-30-FEG scanning electron microscope at an accelerating voltage of 20 kV. TEM studies of the catalysts were performed on a Philips CM12 instrument operating at 120 kV, directly interfaced with a computer for real-time image processing. The specimens were prepared by grinding the powder samples in an agate mortar, suspending and sonicating them in isopropanol, and placing a drop of the suspension on a holey coated carbon copper grid. After evaporation of the solvent, the specimens were introduced into the microscope column.

Raman scattering was measured using a microscope (Olympus BX40, ×50 objective) coupled to a double monochromator (Jobin Yvon Ramanor U-1000) and a photomultiplier (Hamamatsu R943-02) operating in photon-counting mode. Care was taken to minimize heating of the sample by choosing low laser-power (3 mW at its surface). An acquisition time of 30 s was used in order to obtain a sufficient S/N ratio. Spectra were recorded in the range of 100–3,350 cm^−1^ and normalized. Lorentzian bands, superimposed to a constant background, were used to fit the spectra. The center frequency, FWHM and intensity of the bands were chosen by a least-square best-fit method using a commercially available spectroscopic analysis software package. The relative intensities were calculated as integrated-intensity ratios. Further details about instrumentation utilized and spectra-fitting procedure are reported elsewhere [14].

### 2.3. Sensing Tests

**Figure 1.**
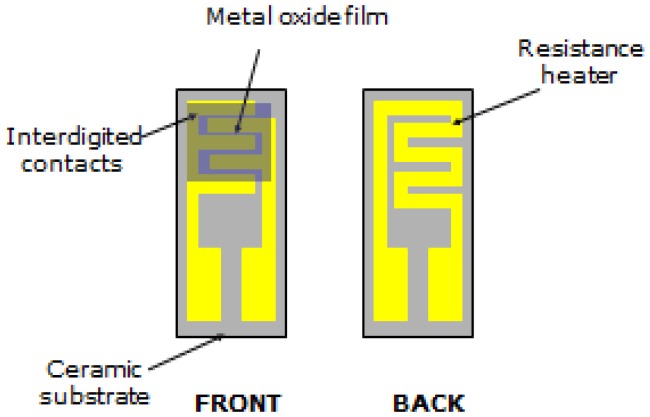
Sensor device.

To fabricate the sensor device, a thick film was deposed by painting an aqueous paste of the composites synthesized on a ceramic substrate (alumina, 6 × 3 mm^2^ sized) supplied with comb-like Pt electrodes and a Pt heater (Figure 1).

**Figure 2.**
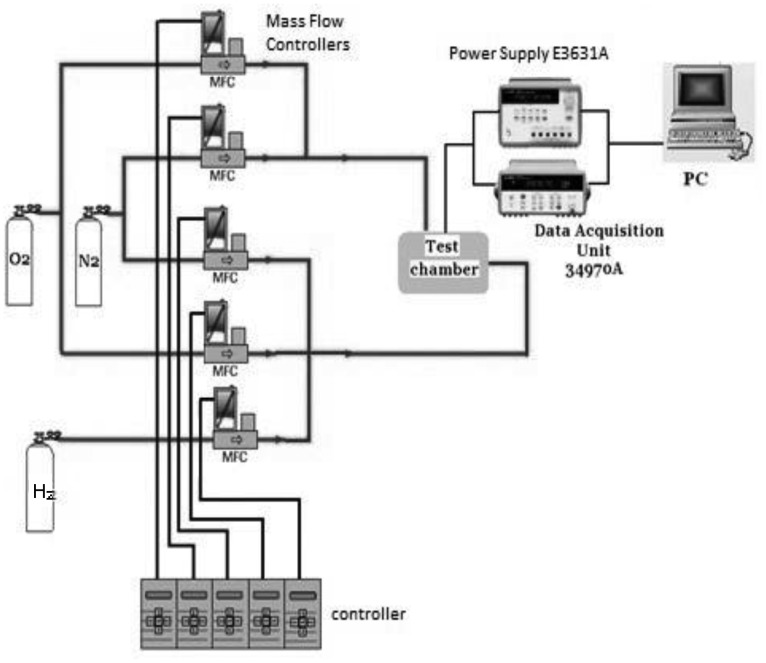
Measurement setup.

Sensing tests were carried out in a home-made apparatus (Figure 2), composed of a stainless test chamber, where the sensor was allocated, and connected to appropriate gas and power supplies.

The sensing devices was interfaced with a PC, which allowed fixing and controlling the working temperature (in the range 50–150 °C), and measuring the resistance values of the sensor under H_2_/Air mixtures flow (100 mL/min).

The response to H_2_ is defined as follows:

(2) $$\text{Sensor response}=\left[\left(\frac{R-R_{0}}{R_{0}}\right)\times 100\right]$$

where R_0_ is the resistance baseline in air and R the resistance under different hydrogen concentrations.

## 3. Results and Discussion

### 3.1. Preparation and Characterization Studies

**Scheme 1.**
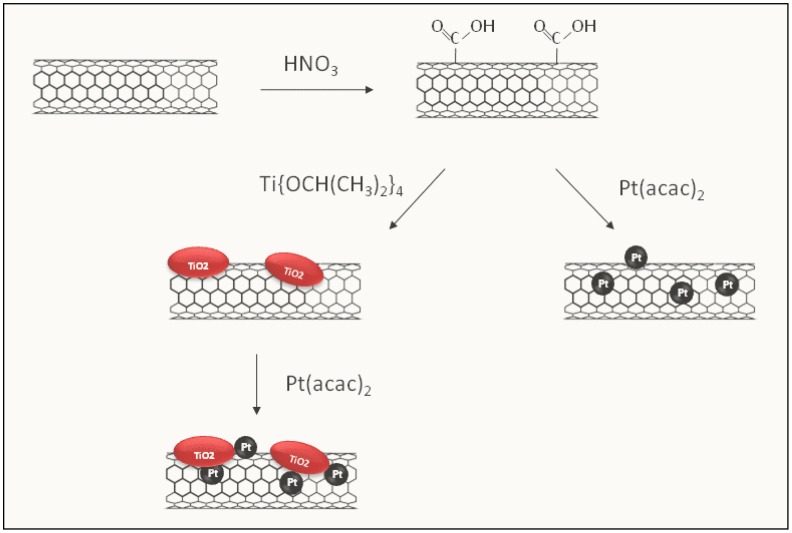
Steps in the preparation of composite samples.

A simple and efficient sol-gel route has been used to synthesize the hybrid composites (see Scheme 1). In a first step, the functionalization of MWCNTs by a chemical treatment at moderate temperature has been carried out. Functionalization provided the formation of suitable attachment sites for Ti and Pt. To prepare the 2-component TiO_2_/MWCNTs and Pt/MWCNTs composites, the functionalized MWCNTs have been then dispersed in a solution of the Ti precursor in isopropanol and of Pt precursor in acetone, respectively, with appropriate concentration. Finally, TiO_2_/MWCNTs has been used as starting material to prepare the 3-component Pt-TiO_2_/MWCNTs composite.

**Figure 3.**
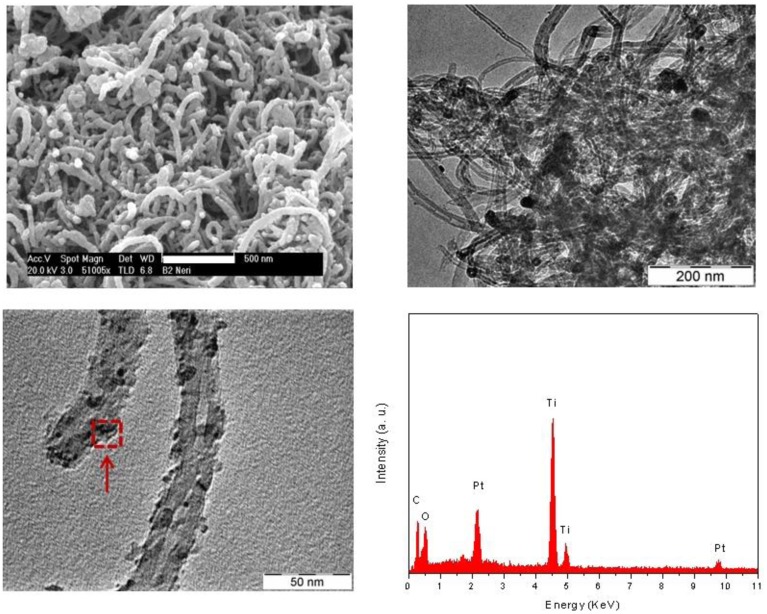
Representative electron microscopy images and EDX analysis of the investigated composites.

The morphology and microstructure of the hybrid composites have been elucidated by electron microscopy. Representative SEM and TEM images are presented in Figure 3. The composite samples are rather heterogeneous, consisting of small titanium oxide and Pt particles dispersed in the nanotube network. However, the single components are intimately mixed together. Indeed, at a closer examination, it appears that nanotubes are partially covered by Pt and TiO_2_ grafted on the nanotube walls. EDX analysis of the region indicated by the arrow in the TEM micrograph clearly demonstrates this by evidencing the presence of Pt, Ti, C and O.

The microscopy characterization suggests then that Pt nanoparticles are well dispersed on the TiO_2_ grains and carbon nanotube support without noticeable agglomeration. This is probably due to the surface functional groups on the functionalized MWCNTs, such as the carboxylic (–COOH) and carbonyl (–C=O) groups, which may help the dispersion of metal nanoparticles, providing furthermore a high level of interaction between the Pt, TiO_2_ and carbon nanotubes.

**Figure 4.**
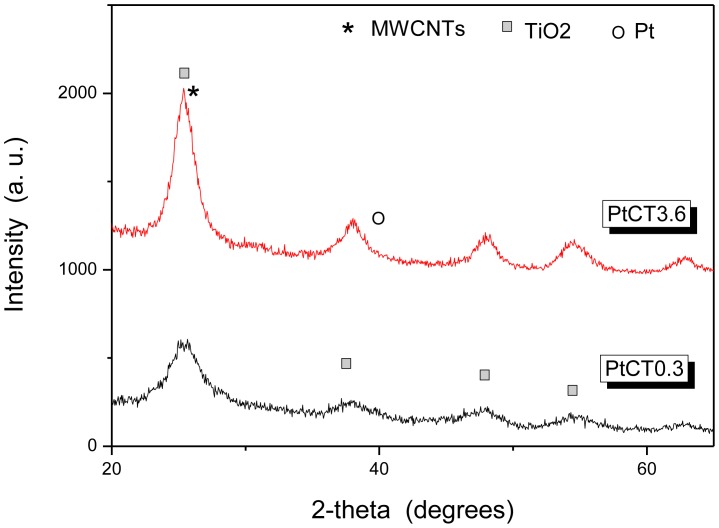
XRD analysis of the ternary Pt-TiO_2_/MWCNTs nanocomposites.

The crystalline phases of the hybrid samples have been analyzed by X-ray powder diffraction, and the result is shown in Figure 4. It can be observed that the crystalline constituent of these samples is primarily the anatase TiO_2_ phase (JCPDS 21-1272). The main peak of MWCNTs cannot longer be discerned in the pattern of sample PtCT3.6, because it is covered by the much more intense peak of titania, while it is discernible in the sample PtCT0.3 where the intensity of titania peaks is lower. This result appears contradictory, because the former sample contain less TiO_2_ with respect to the latter one. However, the smaller intensity of the TiO_2_ peaks on the PtC0.03 sample could be due to a lower degree of cristallinity of the titania phase. The average size of titania crystallites is estimated from the (200) anatase peak at 2θ = 48.0 degree by using the Scherrer equation. The values obtained are in the range between 3 and 5 nm, comparable, within the experimental error, with the size range resulting from TEM observation (4−8 nm). The peaks that can be indexed to Pt in the characteristic face centered cubic (fcc) crystal structure, with the three major peaks at 39.8, 46.2 and 67.41 corresponding to the Pt (111), (200) and (220) planes, respectively, are instead not observed. This suggests that Pt particles presents on the composite samples are amorphous and/or have very small size.

The above characterization data are in good agreement with a more detailed investigation, including a deep Raman analysis, reported elsewhere [9].

### 3.2. Hydrogen Sensing Tests

Recently, inorganic/CNT hybrids have emerged as a new and very promising class of functional materials, with outstanding potential for a wide range of applications concerning energy and the environment, that combine the multiphase characteristics of nanocomposites with the synergistic functions of hybrid frameworks. Composites with WO_3_, TiO_2_ and SnO_2_ as the metal oxide phase supported on carbon nanotubes [15–17] are some examples of hybrid materials used as sensing layers in resistive sensors. The main role of the carbon nanotubes is to stabilize highly dispersed metal or metal oxide nanoclusters, resulting in a high specific surface area [18]. However, in some cases, hybrid composites show improved properties other than the higher surface area, such as higher electron conductivity due to the presence of CNTs. The increase in the sensing capability of the hybrid metal-metal oxide/CNTs composites was therefore commonly ascribed to the synergic effect of the high electron conductivity of the carbon nanostructure with the sensing and catalytic properties of the inorganic constituents material.

In a previous paper, we have used the synthesized Pt-TiO_2_/MWCNTs hybrid composites to fabricate hydrogen sensors able to operate at near room temperature in the presence of high H_2_ concentrations (up to 100%) in a nitrogen atmosphere. These sensors were specifically designed to monitor the concentration of hydrogen in the feed of PEM fuel cells. Here, these sensors are investigated to detect hydrogen in air. Hydrogen is a flammable gas when present in concentrations higher than 4% in air, than the continuous monitoring of hydrogen leaks in a fuel cell during the various steps of H_2_ production and use is an essential issue for safety reasons.

**Figure 5.**
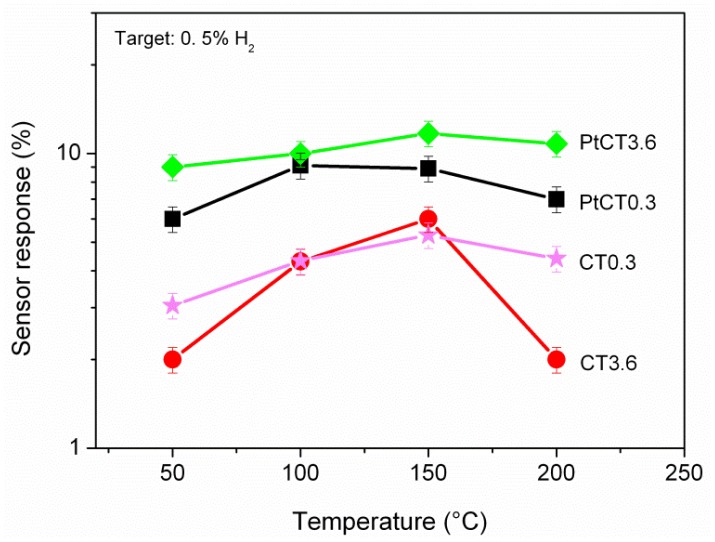
Response of the sensors investigated at different operating temperatures.

The sensors were fabricated by painting an aqueous paste of the hybrid nanocomposites with different C/Ti molar ratio on the sensor substrates, so to obtain thick Pt/TiO_2_/MWCNTs films. Preliminary tests were carried out in order to establish the optimal operating temperature. Figure 5 shows clearly that the higher responses can be obtained at around 100–150 °C. In order to compare all sensors under the same experimental conditions, in the following, sensing data are referred to the optimal operating temperature of 150 °C.

**Figure 6.**
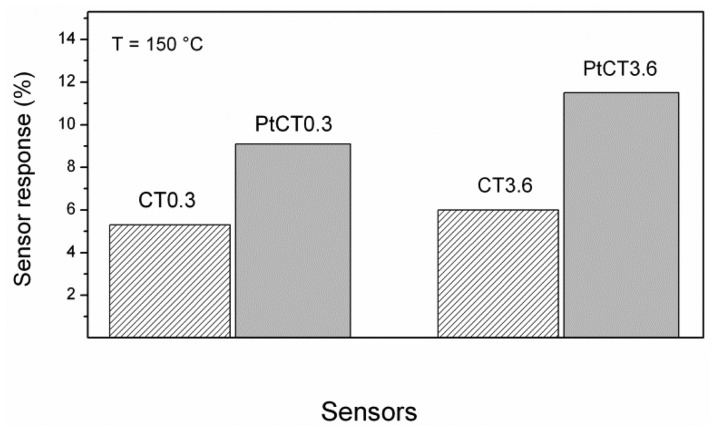
Comparison of the response of composite sensors with different C/Ti ratios and Pt loading to pulses of 0.5% of H_2_ in air of the duration of 250 seconds.

During the previous investigation in nitrogen atmosphere, it has been found that an optimal C/Ti ratio is necessary to provide a high response [9]. The bar graph reported in Figure 6 indicates that in air and at the reference temperature the response is almost independent on the C/Ti ratio, depending instead on the presence of platinum. This can be explained assuming that a different sensing mechanism occurs under air atmosphere, where likely the surface reaction between hydrogen and oxygen, catalyzed by platinum, play the dominant role. A mechanism which takes into account the key role of platinum will be proposed and discussed below.

**Figure 7.**
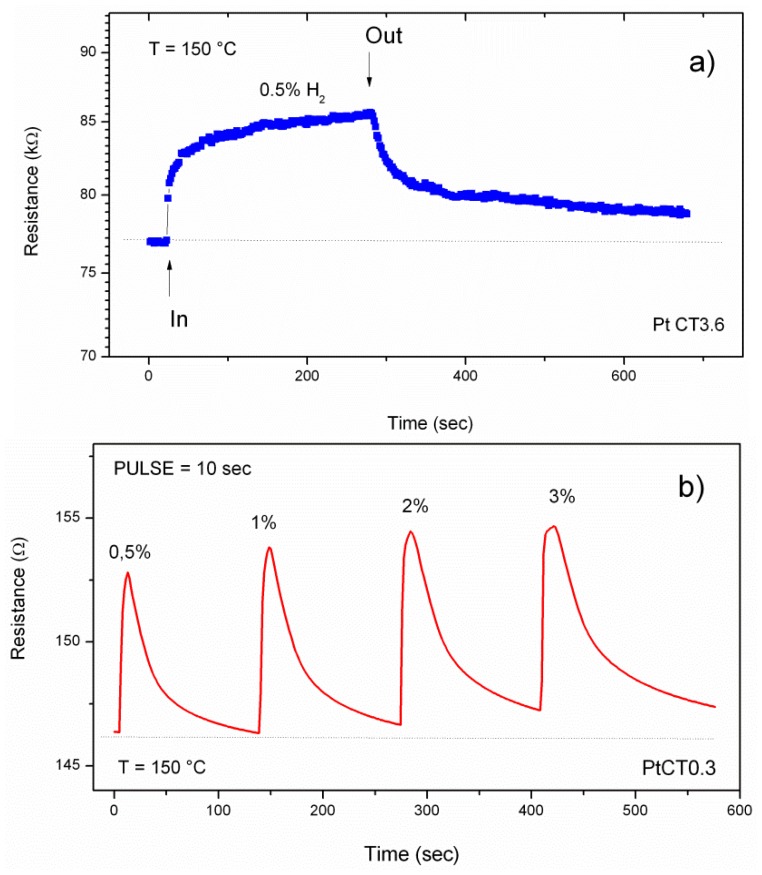
Resistance change at 150 °C of sensors to hydrogen pulses. (**a**) PtCT3.6; (**b**) PtCT0.3 to hydrogen pulses of the duration of 10 seconds.

The transient response of the PtCT0.3 sensor applying a step H_2_ concentration of 0.5% is shown in Figure 7(a). The sensor reaches the steady state response within 150–200 seconds, whereas a longer time is necessary for complete recovery when the H_2_ is turned off. For a leak sensor, the response speed should be as fast as possible. Therefore, we evaluated the response of sensors to faster pulses of H_2_, in the concentration range between 0.5–3%. The dynamic response of the Pt-TiO_2_/MWCNTs sensor to pulses of H_2_ at different concentrations, of only 10 seconds duration, are reported in Figure 7(b).

**Figure 8.**
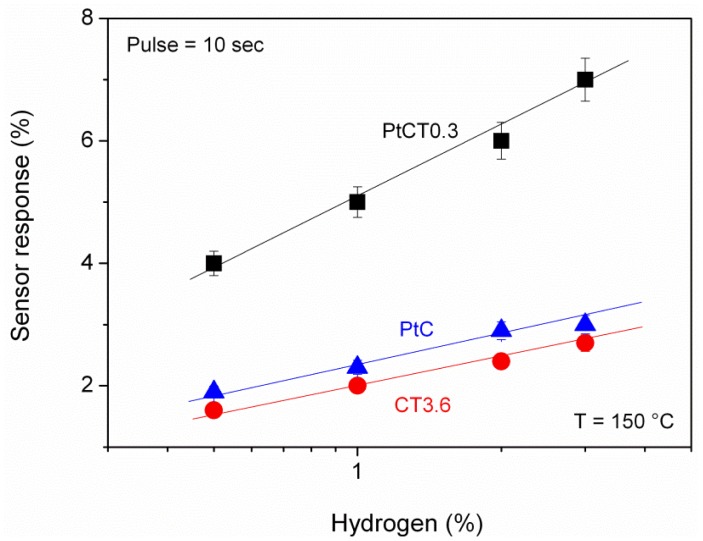
Calibration curves of different composite sensors.

Figure 8 reports the calibration curve for some sensors, in the hydrogen concentration range in air between 0.5–3% in volume, obtained with hydrogen pulses of the duration of 10 seconds. The solid line represents a weighted least squares fit of the experimental data.

The comparison of the sensor response of Pt/MWCNTs, TiO_2_/MWCNTs and Pt-doped TiO_2_/MWCNTs sensors to H_2_ clearly indicated a catalytic promoter effect of the platinum. This is further confirmed by the fact that pure MWCNTs, differently to the Pt-doped MWCNTs (sample PtC), are insensitive to H_2_. Pt acts as a catalytic additive improving the reactions which occur on the surface of the sensing layer. Furthermore, the action of platinum is enhanced in the presence of the metal oxide. The 3-component hybrid platform as a sensing element is in fact superior to either of its constituent components.

Baseline drift and response stability are also important features for the practical applications of gas sensors. During repeated pulses, the short-term stability in the baseline and response of the sensor has been investigated. The results indicate that the sensor has good stability and reliability.

On the basis of the reported results, a plausible mechanism can be formulated, attributing the enhanced performance of Pt-loaded sensors to a “spill-over effect” driven by Pt particles combined with their high catalytic activity. Pt is in a metallic state and highly dispersed in forms of small particles on the surface of the supporting metal oxide/carbon nanotubes composite system. In such a form, under air, Pt is able to facilitates the dissociation of oxygen. Then, the surface of Pt-loaded composites is covered by a higher amount of oxygen adsorbed species. This is supported by the measured more elevated baseline resistance for the Pt-doped semiconducting sensors. In the presence of hydrogen, Pt also dissociates hydrogen molecules, transferring H atoms onto the titania surface, as previously reported [9]. Furthermore, it is known that Pt particles exhibit a high chemical activity for H_2_ oxidation [19]. On the basis of these findings, it can be supposed that the higher amount of reactive oxygen ions consequently determines the improved sensor performance, through the oxidation of chemisorbed hydrogen atoms by oxygen ions catalyzed by Pt.

## 4. Conclusions

Pt-TiO_2_/MWCNTs hybrid composites have been synthesized by a simple sol-gel route. Sensing tests have shown that these Pt-TiO_2_/MWCNTs devices are sensitive to low hydrogen concentrations in air. Pt exhibited a promoter effect, likely favoring the dissociation of hydrogen molecules at low temperature and activating the surface reaction between adsorbed hydrogen and oxygen species. The results obtained suggest then that the sensing performance of the hybrid nanostructure sensor could be attributed to: (i) the increase in the specific surface area of hybrid nanostructures; (ii) the effective electron transfer between TiO_2_ grain and MWCNTs; (iii) the catalytic action of platinum. Experiments with different metal oxides, carbon nanostructures and/or different preparation procedures are in progress in order to further understand the sensing properties of these composite materials and fabricate hydrogen sensors with enhanced performances.

## Figures and Tables

**Table 1.** Chemical composition of the investigated samples.

**Sample**	**C/Ti molar ratio**	**Pt (wt%)**	**TiO_2_ (wt%)**	**MWCNTs (wt%)**
CT0.3	0.3	-	93.1	4.9
CT 3.6	3.6	-	63.7	34.3
PtC	-	2.0	-	98
PtCT0.3	0.3	2.0	93.1	4.9
PtCT3.6	3.6	2.0	63.7	34.3
